# The Importance of Preserving the Teeth

**Published:** 1852-04

**Authors:** 


*The Importance of Preserving the Teeth.* By John Linn, *Dentist.* Portsmouth, Va.

This is a little popular treatise, and will, we have no doubt, do good. It is a question of ethics, however, how nearly such publications approach advertisements. Dentists should be on their guard in this matter. It is well to give the people information, and in one sense all medical books may be considered advertisements. The dividing line here is as sharp as a razor's edge. He must walk cautiously, who will venture on it.

